# Evaluating Poly‐D,L‐Lactic Acid for Lower Eyelid Rejuvenation: Efficacy and Safety

**DOI:** 10.1111/jocd.70058

**Published:** 2025-02-28

**Authors:** Jovian Wan, Inneke Jane Hidajat, Hugues Cartier, Sebastien Garson, Carlos Bautzer, Lucas Basmage Machado, Patricia Leite, Kyu‐Ho Yi

**Affiliations:** ^1^ Medical Research Inc. Wonju Korea; ^2^ Universitas Katolik Indonesia Atma Jaya Fakultas Kedokteran Dan Ilmu Kesehatan Indonesia; ^3^ Centre Médical Saint Jean Arras France; ^4^ Cabinet Médical Senlis France; ^5^ Lifestyle Clinic Sao Paulo Brazil; ^6^ Synergie Clinic Campo Grande Brazil; ^7^ Patricia Leite Clinic Belo Horizonte Brazil; ^8^ Division in Anatomy and Developmental Biology, Department of Oral Biology, Human Identification Research Institute, BK21 FOUR Project Yonsei University College of Dentistry Seoul Korea; ^9^ You & I Clinic (Mokdong) Seoul Korea

**Keywords:** infraorbital hollows, lower eyelid rejuvenation, nasojugal groove, poly‐L‐lactic acid, tear trough deformity

## Abstract

**Background:**

Injectable fillers like PDLLA offer a novel approach to addressing lower eyelid rejuvenation. Despite their growing popularity, comprehensive studies on newer products like PDLLA are limited.

**Aims:**

This study evaluates the efficacy and safety of PDLLA (Juvelook, VAIM Inc., Seoul, Korea) for treating tear trough deformities in Korean women, aiming to provide a long‐term, minimally invasive solution for periorbital rejuvenation.

**Materials & Methods:**

A selected group of Korean women exhibiting tear trough deformities underwent treatment with PDLLA. Patients were assessed pre‐treatment and followed up at 1 week, 1 month, and 6 months post‐treatment, with outcomes measured by independent clinicians using the Hirmand classification and patient satisfaction scores.

**Results:**

PDLLA demonstrated significant improvements in tear trough deformities with high patient satisfaction and minimal adverse effects. Follow‐up results showed sustained improvements and no severe complications, indicating a safe profile for PDLLA.

**Discussion:**

The study highlights PDLLA's potential advantages over traditional fillers, including longer‐lasting effects and enhanced collagen production, suggesting it as a superior option for those seeking durable aesthetic improvements.

**Conclusion:**

PDLLA is an effective and safe treatment for periorbital rejuvenation in Korean women, providing lasting benefits and high patient satisfaction, warranting further comparative studies for broader applications.

## 1 Introduction

The application of injectable fillers for facial rejuvenation has witnessed substantial advancement and increased popularity in recent years, largely due to their minimally invasive nature and relatively swift recovery periods. Among the myriad of facial regions addressed by these treatments, the lower eyelid area presents unique challenges attributable to its intricate anatomical features and the pronounced age‐related alterations it undergoes.

Aging induces multiple changes in the lower eyelid, which significantly impacts its aesthetic appearance. Common age‐related concerns include tear trough deformities, infraorbital hollows, nasojugal grooves, dark circles, and mild periorbital edema. These manifestations are often exacerbated by a combination of fat loss, skin thinning, and modifications in the underlying bone structure. The delicate nature of the lower eyelid's skin, coupled with the proximity of critical vascular and neural structures, further complicates the effective management of these issues with injectable fillers.

Historically, hyaluronic acid (HA) and calcium hydroxyapatite (CaHA) have been predominant choices for addressing lower eyelid concerns. HA fillers are valued for their immediate efficacy and safety profile but may occasionally result in complications such as the Tyndall effect or prolonged swelling. Conversely, CaHA provides longer‐lasting effects but can be more challenging to administer and may not always yield optimal aesthetic results.

Recent innovations have introduced PDLLA (Juvelook, VAIM Inc., Seoul, Republic of Korea), a poly‐D,L‐lactic acid product, as a promising alternative. PDLLA is notable for its collagen‐stimulating properties, which promote gradual collagen production, potentially offering more natural and sustained improvements in facial volume and texture. Preliminary studies indicate that PDLLA's mechanism of action may contribute to longer‐lasting results with potentially fewer adverse effects compared to traditional fillers.

Despite these advancements, current research on PDLLA remains limited in terms of diverse patient populations, longer follow‐up periods, and comparative studies with other fillers. This study seeks to address these gaps by presenting findings specific to lower eyelid rejuvenation using PDLLA.

This study explores a novel approach utilizing PDLLA for lower eyelid rejuvenation, particularly for Asian patients. The proposed method aims to simplify the treatment process while optimizing outcomes. By examining PDLLA's unique attributes and its potential advantages, this research seeks to provide a new, effective technique for improving the appearance of the lower eyelid region.

## 2 Methods

### 2.1 Patient Selection

Candidates for this study were individuals presenting with tear trough deformities, infraorbital hollows, nasojugal grooves, dark circles, and mild eye bags. Patients exhibiting mid‐facial volume loss were first treated with HA filler administered to the supra‐periosteal layer at least 1 week prior to the lower eyelid injection. Exclusion criteria included excessive lower eyelid laxity, recent lower eyelid surgery or filler injections within the past year, allergies to filler components, a tendency for keloid formation pregnancy or breastfeeding, coagulation disorders, the use of anticoagulant medications, unrealistic expectations, and any medical or psychological conditions unsuitable for injection.

### 2.2 Preinjection Protocol

Upon meeting the inclusion criteria, patients provided informed consent, underwent facial cleansing, and had preinjection photographs taken. A topical anesthetic cream was then applied to the lower eyelid and cheek areas and left in place for 15–20 min to ensure effective local anesthesia.

### 2.3 Filler Preparation and Injection Technique

The injectable PDLLA (Juvelook, VAIM Inc., Seoul, Korea) was reconstituted with sterile water (9 ml) for injection according to the severity of the volume deficit. Patients' tear trough deformities were classified according to Hirmand's [1] framework, which delineates volume loss into three distinct classes:

- Class I: Volume loss confined to the medial tear trough area, potentially extending to mild flattening of the central cheek.
- Class II: Volume loss affecting both the medial and lateral orbital areas, with moderate volume deficiency extending to the medial cheek and flattening of the central upper cheek.
- Class III: Extensive volume loss with a full circumferential depression around the orbital rim, from medial to lateral.

### 2.4 Injection Procedure

Patients were positioned at a 45° angle. After sterilization with alcohol, a 23G cannula was inserted at a point 10‐mm inferolateral to the lateral canthus. The cannula was advanced horizontally and superficially in the upper subcutaneous layer to ensure accurate placement. Using the fanning technique, PDLLA (Juvelook, VAIM Inc., Seoul, Korea) was deposited in small droplets, with a maximum volume of 1 mL per side. Care was taken to avoid concentrating the product in any single area. Video S1 demonstrates the injection technique used.

### 2.5 Postinjection Care

After the injection, the treatment area was gently massaged for 5–10 s. Patients were advised against home massage to minimize complications.

### 2.6 Outcome Measures

Evaluations involved a blinded, independent reassessment of tear trough deformities using the Hirmand [1] classification system by two clinicians. Follow‐up assessments were conducted 1 week, 1 month, and 6 months after treatment. Common side effects such as bruising, edema, and erythema were monitored, with any serious adverse effects documented. Detailed patient satisfaction measures included visual analog scales (VAS) to quantify satisfaction, with scores ranging from 1 (least satisfied) to 10 (most satisfied).

### 2.7 Cases

#### 2.7.1 Case 1

**FIGURE 1:**
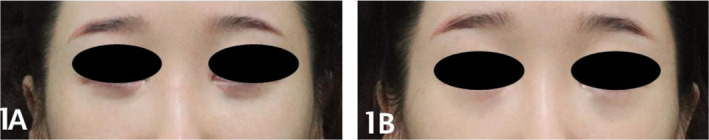
Pretreatment image of a 41‐year‐old Korean woman with Fitzpatrick skin type 3, presenting with a Class II tear trough deformity. The image shows volume loss extending from the medial to the lateral orbital areas and moderate flattening of the central (A). Six months posttreatment image following the administration of 1 mL each side of PDLLA in a single session. The tear trough deformity demonstrates complete resolution, maintaining the Class I classification with sustained improvement. The patient reports high satisfaction, minimal bruising, and no serious adverse effects (B).

A 41‐year‐old Korean woman with Fitzpatrick skin type 3 initially presented with a Class II tear trough deformity, characterized by volume loss extending from the medial to the lateral orbital areas and moderate flattening of the central cheek (Figure 1A). She was treated with 1 mL of PDLLA on each side in a single session. One week posttreatment, an independent, blinded reassessment by two clinicians using the Hirmand classification system confirmed complete resolution of the tear trough deformity, with a significant reduction in hollowness and enhanced volume. Six months after treatment (Figure 1B), the deformity remained fully resolved, demonstrating sustained improvement and high patient satisfaction. Minimal bruising was reported, and no serious adverse effects were observed throughout the follow‐up period.

#### 2.7.2 Case 2

**FIGURE 2:**
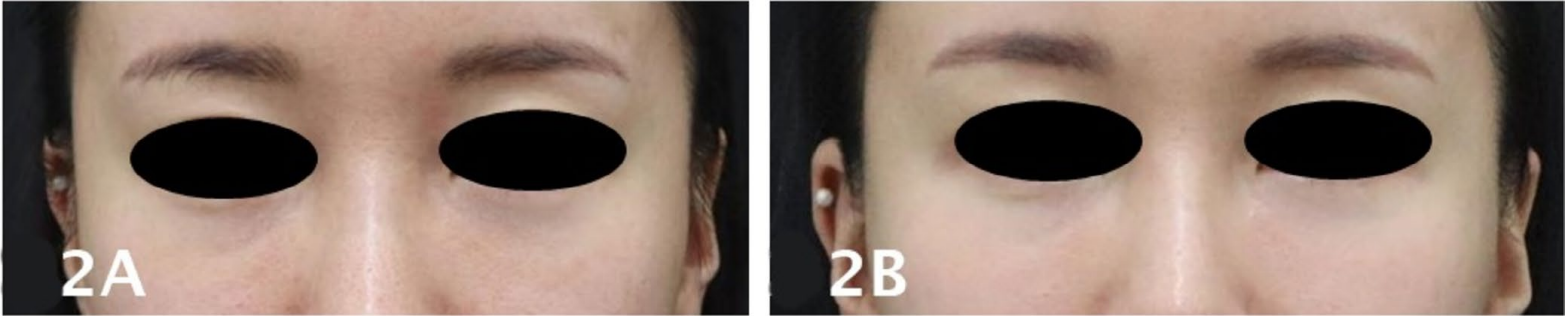
Pretreatment photograph of a 36‐year‐old Korean woman with a Class I tear trough deformity (A), showing volume loss limited to the medial tear trough with minimal flattening of the central cheek. Six months posttreatment photograph of the same patient (B), demonstrating complete resolution of the tear trough deformity following 1 mL each side of PDLLA treatment.

A 36‐year‐old Korean woman with Fitzpatrick skin type 3 presented with a Class I tear trough deformity, characterized by volume loss limited to the medial tear trough with minimal flattening of the central cheek (Figure 2A). She received 1 mL of PDLLA on each side in a single session. Six months posttreatment (Figure 2B), an independent, blinded reassessment by two clinicians using the Hirmand classification system indicated complete resolution of the tear trough deformity, with no detectable hollowness or volume loss. The patient expressed high satisfaction with the results, reported minimal bruising, and experienced no serious adverse effects throughout the follow‐up period.

#### 2.7.3 Case 3

**FIGURE 3:**
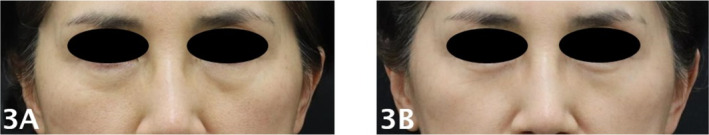
Pretreatment photograph of a 54‐year‐old Korean woman (A) with a Class III tear trough deformity, showing extensive volume loss and pronounced hollowness extending from the medial to the lateral orbital areas, along with significant flattening of the central cheek. Six months posttreatment photograph of the same patient (B), demonstrating improvement to a Class II tear trough deformity following 0.4 mL of PDLLA treatment, with noticeable reduction in hollowness and enhanced volume.

A 54‐year‐old Korean woman with Fitzpatrick skin type 3 initially presented with a Class III tear trough deformity, characterized by extensive volume loss extending circumferentially along the orbital rim (Figure 3A). She was treated with 1 mL of PDLLA in a single session. One week after treatment, an independent, blinded reassessment by two clinicians using the Hirmand classification system showed improvement to a Class II deformity, with a notable reduction in hollowness and enhanced volume. By 6 months (Figure 3B), the tear trough deformity remained classified as Class II, demonstrating sustained improvement. The patient reported a significant increase in confidence regarding her appearance. Mild swelling was observed initially but resolved within 48 h. No serious adverse effects were noted throughout the follow‐up period.

#### 2.7.4 Case 4

**FIGURE 4:**
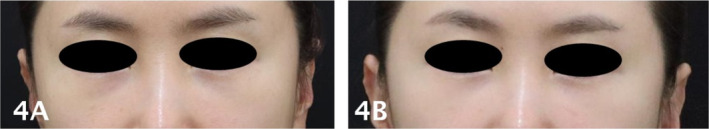
Pretreatment photograph of a 51‐year‐old Korean woman with a Class I tear trough deformity (A), showing volume loss limited to the medial aspect of the tear trough with minimal flattening of the central cheek. Six months posttreatment photograph of the same patient (B), demonstrating complete resolution of the tear trough deformity following 1 mL each side of PDLLA treatment.

A 38‐year‐old Korean woman with Fitzpatrick skin type 3 initially presented with a Class II tear trough deformity, characterized by volume loss extending from the medial to the lateral orbital areas and moderate flattening of the central cheek (Figure 4A). She received 1 mL of PDLLA in each side a single session. One week after treatment (no figure available), an independent, blinded reassessment by two clinicians using the Hirmand classification system revealed improvement to a Class I deformity, with a noticeable reduction in hollowness and enhanced volume. Six months posttreatment (Figure 4C), the tear trough deformity remained consistently classified as Class I, showing sustained improvement and high patient satisfaction. Mild bruising and swelling were observed initially but resolved within 48 h. No serious adverse effects were reported throughout the follow‐up period.

## 3 Discussion

The present study aimed to evaluate the efficacy and safety of injectable PDLLA for treating tear trough deformities in Korean women with Fitzpatrick skin type III. The results suggest that PDLLA is a promising option for nonsurgical lower eyelid rejuvenation, demonstrating significant improvements in tear trough appearance with a relatively low incidence of adverse effects. However, as this is a preliminary evaluation of PDLLA, several limitations must be considered when interpreting these findings, which will inform the direction of future research.

Our findings illustrate that PDLLA effectively addresses various degrees of tear trough deformities, as classified by the Hirmand system. Each patient in the study demonstrated marked improvements after a single session of treatment. For example, Case 1, with a Class II tear trough deformity, showed a substantial reduction in hollowness and enhanced facial aesthetics following 1 mL each side of PDLLA. Similarly, Case 2, classified as Class I, achieved complete resolution with 1 mL of PDLLA. Follow‐up assessments at 6 months confirmed that these aesthetic benefits were sustained over time.

Case 3 involved a patient with a Class III tear trough deformity, presenting with extensive volume loss along the orbital rim (Figure 3A). The patient responded well to a 1 mL of each side injection of PDLLA, showing notable improvement to a Class II deformity 1 week after treatment and sustained benefits at the 6‐month follow‐up (Figure 3B). Case 4, a Class II patient, also demonstrated significant enhancements with 1 mL each side of PDLLA, further supporting the efficacy of this treatment approach.

The safety profile of PDLLA observed in this study is consistent with existing literature. Common side effects such as bruising, edema, and erythema were transient and resolved within a few days, aligning with outcomes reported in previous studies. These side effects were generally mild and self‐limiting, reflecting the localized nature of the treatment and the body's natural healing processes [2, 3, 4, 5, 6]. Importantly, no serious adverse effects were reported within the 6 months of follow‐up. The minimal incidence of severe complications, such as infections or vessel embolisms, underscores the overall safety of PDLLA, suggesting it is a well‐tolerated option for aesthetic treatments.

When compared to other dermal fillers, such as HA, PDLLA offers several notable advantages. HA fillers, while effective for immediate volume restoration and contour enhancement, typically provide shorter‐lasting results. Patients often require more frequent touch‐ups to maintain their desired aesthetic outcomes, which can lead to increased treatment costs and more frequent clinic visits [7, 8, 9]. In contrast, PDLLA has a distinct mechanism of action; it stimulates the body's own collagen production, leading to gradual and sustained improvements in skin quality and volume. This biological stimulation of collagen helps to rebuild and strengthen the skin's structure over time, offering longer‐lasting results with fewer maintenance sessions [10, 11, 12]. While preclinical animal studies and preliminary clinical reports suggest that PDLLA fillers provide longer‐lasting effects compared to HA fillers due to their biostimulatory properties, definitive clinical trials comparing their durability directly are limited. To date, no large‐scale, randomized controlled trials have confirmed that PDLLA fillers unequivocally yield more durable outcomes than HA fillers [12, 13]. Addressing this gap in the literature would require robust clinical studies involving diverse patient populations and extended follow‐up periods to establish PDLLA's superiority in terms of longevity and clinical effectiveness. This limitation underscores the need for future comparative research.

Moreover, the long‐term structural benefits provided by PDLLA can enhance the overall appearance and health of the skin, contributing to a more youthful and natural‐looking result. This contrasts with HA fillers, which may require more frequent reinjections to maintain volume and contour [14]. The ability of PDLLA to induce collagen production means that, over time, the treated area becomes more resilient and aesthetically pleasing, offering a sustainable and effective solution for addressing tear trough deformities [2, 11, 12, 13]. This aspect of PDLLA highlights its potential as a superior alternative for patients who prioritize long‐term results and a reduced need for ongoing treatments [15, 16, 17, 18]. Despite these promising results, the study is limited by its small sample size and lack of a control group, which restricts the generalizability of the findings. A larger cohort would provide more robust data, allowing for a better understanding of the range of outcomes in different patient populations. Additionally, the lack of a control group limits our ability to directly compare the efficacy and safety of PDLLA to other fillers, such as HA, in treating tear trough deformities. A control group would strengthen the conclusions by providing a benchmark against which PDLLA's effects can be measured. Furthermore, the follow‐up period in this study was relatively short (6 months). Although the results at this time point were promising, longer term studies are necessary to assess the durability of the effects and to understand the long‐term safety profile of PDLLA in the periorbital region. Given that collagen‐stimulating fillers like PDLLA work through gradual tissue remodeling, it is important to track patients for a longer duration to fully evaluate the sustained benefits and any potential late‐onset complications.

Incorporating detailed patient satisfaction measures would also enhance the study. While objective outcomes such as volume restoration and skin quality improvement are essential, patient‐reported outcomes are crucial for understanding the overall effectiveness of the treatment. Future studies should integrate validated satisfaction questionnaires to better capture the impact of PDLLA on patients' quality of life and their perceived aesthetic improvements.

The patient population in this study was limited to Korean women with Fitzpatrick skin type III, which may not reflect the full spectrum of individuals seeking treatment for tear trough deformities. A more diverse population, including individuals with different skin types and ethnic backgrounds, would provide more comprehensive data on the safety and efficacy of PDLLA in a broader patient group. Further research is needed to explore how PDLLA performs across various demographics.

In conclusion, injectable PDLLA has proven to be an effective and safe option for addressing tear trough deformities in Korean women with Fitzpatrick skin type III. The treatment led to significant aesthetic improvements with minimal adverse effects, supporting PDLLA as a viable alternative to other fillers. However, due to the limitations of this study, including a small sample size, a lack of a control group, and a short follow‐up, further studies with larger cohorts, extended follow‐up periods, and more detailed patient‐reported outcomes are necessary to confirm these findings and refine treatment approaches for optimal patient outcomes.

## Author Contributions

All authors have reviewed and approved the article for submission. Conceptualization – Jovian Wan, Kyu‐Ho Yi. Study design – Jovian Wan; Inneke Jane Hidajat. acquisition of data – Hugues Cartier; Sébastien Garson; writing – original draft preparation – Jovian Wan, Kyu‐Ho Yi, Carlos Bautzer, Lucas Basmage Machado, Patricia Leite. Writing – editing and revision – Jovian Wan, Inneke Jane Hidajat, Hugues Cartier; Sébastien Garson, Lucas Basmage Machado; Patricia Leite. Visualization – Jovian Wan, Kyu‐Ho Yi. supervision – Kyu‐Ho Yi.

## Conflicts of Interest

I acknowledge that I have considered the conflicts of interest statement included in the “Author Guidelines.” I hereby certify that, to the best of my knowledge, no aspect of my current personal or professional situation might reasonably be expected to significantly affect my views on the subject I am presenting.

## Supporting information


**Video S1.** Detailed Demonstration of Injection Technique for Tear Trough Correction.
This video showcases the precise technique used for administering PDLLA fillers to correct tear trough deformities, infraorbital hollows, and mild eye bags, following the classification of volume loss according to Hirmand’s framework. Observe the methodical approach involving the preparation and injection of the filler, using a 23G cannula and the fanning technique to ensure even distribution and minimize complications. This demonstration is critical for practitioners aiming for optimal outcomes in facial aesthetics.

## Data Availability

The data that support the findings of this study are available from the corresponding author upon reasonable request.
